# Allogeneic adoptive cell transfer therapy as a potent universal treatment for cancer

**DOI:** 10.18632/oncotarget.300

**Published:** 2011-06-29

**Authors:** Assaf Marcus, Zelig Eshhar

**Affiliations:** ^1^Department of Molecular Cell Biology, University of California Berkeley; ^2^Department of Immunology, The Weizmann Institute of Science

Adoptive cell transfer (ACT), the transfer of therapeutic lymphocytes to patients, is a promising cancer treatment, with advances seen in patients with melanoma and neuroblastoma in early clinical trials [1, 2]. One type of ACT modality pioneered by our group relies on the use chimeric antigen receptors (CAR) redirected T cells [3, 4]. CARs are typically composed of single chain antibody fragments fused to T cell activating signaling motifs, endowing the T cells with MHC unrestricted tumor specificity. One advantage of CARs over conventional TCRs is that costimulatory domains could be incorporated into the CAR endowing it with supra-physiological potency.

One limitation of ACT is the need to generate tumor specific lymphocytes for each individual patient, which is technically and economically challenging. We therefore sought to develop an approach for universal ACT, which would allow treatment of patients without the need for MHC matching [5]. We reasoned that because of their MHC unrestricted tumor specificity, allogeneic CAR redirected T cells could be used as ‘universal effector cells’ against cancer in all patients. However, allogeneic adoptive transfer faces the problem of the host-versus-graft response (HvG) and the danger of graft-versus-host-disease (GvHD).

We hypothesized that low dose irradiation would delay the rejection of allogeneic T cells and allow them enough time to destroy the tumor on one hand while on the other hand their eventual rejection would prevent the development of GvHD. Using this approach, fully MHC mismatched allogeneic T cells redirected with a HER2/neu specific CAR were able to provide as much therapeutic benefit as syngeneic cells in a model of metastatic disease. We demonstrated that the GvH reactivity of the allogeneic T cells caused extensive proliferation, augmenting the anti-tumor response, but that the allogeneic T cells were indeed eventually rejected and no GvHD developed.

Our second working hypothesis was that modulating lymphocyte migration could potentially further increase therapeutic efficacy of allogeneic ACT. Both the HvG and GvH reactions originate in the lymph nodes where T cells encounter MHC mismatched antigen presenting cells (APC), undergo massive proliferation, and then egress out of the lymph nodes into peripheral organs. By contrast ACT introduces cells directly into the blood, circumventing egress, and allowing the cells to reach the tumor directly. We therefore surmised that blocking lymphocyte egress using the lymphocyte sequestering agent FTY720[6] might inhibit the HvG and GvH reactions to a much greater extent than the graft-versus-tumor (GvT) reaction. Indeed, FTY720 enhanced the efficacy of allogeneic but not syngeneic ACT, such that allogeneic T cells provided superior therapeutic benefit over syngeneic ones with median survival of over one year and no manifestations of GvHD.

In conclusion, our results suggest that MHC mismatched CAR redirected allogeneic T cells could function as ‘universal effector cells’, and could potentially be used as an ‘off-the-shelf’ cellular therapy of cancer. Blocking lymphocyte egress further increased the efficacy of the approach such that allogeneic cells were superior to syngeneic ones. This result conclusively demonstrates that we can harness the GvH reactivity of allogeneic T cells to augment the anti-tumor response without causing GvHD. While this work provides only a proof of principle, we strongly believe that this approach is widely applicable for two main reasons. First, by using other CAR, many different cancers could be targeted of both solid and hematologic malignancies [7]. Second, the HvG and GvH occur independently of the tumor, and since we were able to overcome this problem in our work, the same approach could be used regardless of tumor type. Taken together, our results suggests that with further tuning, allogeneic adoptive cell therapy may become the treatment of choice both because of its obvious technical and economical advantages and due to its greater efficacy.
